# Vgas: A Viral Genome Annotation System

**DOI:** 10.3389/fmicb.2019.00184

**Published:** 2019-02-13

**Authors:** Kai-Yue Zhang, Yi-Zhou Gao, Meng-Ze Du, Shuo Liu, Chuan Dong, Feng-Biao Guo

**Affiliations:** Centre for Informational Biology, School of Life Science and Technology, University of Electronic Science and Technology of China, Chengdu, China

**Keywords:** Vgas, virus gene prediction, function annotation, novel genes, joint application of multiple programs

## Abstract

The in-depth study of viral genomes is of great help in many aspects, especially in the treatment of human diseases caused by viral infections. With the rapid accumulation of viral sequencing data, improved, or alternative gene-finding systems have become necessary to process and mine these data. In this article, we present Vgas, a system combining an *ab initio* method and a similarity-based method to automatically find viral genes and perform gene function annotation. Vgas was compared with existing programs, such as Prodigal, GeneMarkS, and Glimmer. Through testing 5,705 virus genomes downloaded from RefSeq, Vgas demonstrated its superiority with the highest average precision and recall (both indexes were 1% higher or more than the other programs); particularly for small virus genomes (≤ 10 kb), it showed significantly improved performance (precision was 6% higher, and recall was 2% higher). Moreover, Vgas presents an annotation module to provide functional information for predicted genes based on BLASTp alignment. This characteristic may be specifically useful in some cases. When combining Vgas with GeneMarkS and Prodigal, better prediction results could be obtained than with each of the three individual programs, suggesting that collaborative prediction using several different software programs is an alternative for gene prediction. Vgas is freely available at http://cefg.uestc.cn/vgas/ or http://121.48.162.133/vgas/. We hope that Vgas could be an alternative virus gene finder to annotate new genomes or reannotate existing genome.

## Introduction

Because of the tremendous value of in-depth studies of viral genomes for the treatment of human infectious diseases caused by viral infections, many viroinformatics resources, including web servers and databases, have been developed (Sharma et al., 2015). The number of sequenced viral genomes stored in the RefSeq database has increased more than five times from the year 2000 to 2016 with the rapid development of sequencing technologies (Brister et al., 2015). For the investigation of viral genomes, the first and most important step is to annotate genes accurately. Although wet experiments likely represent the most accurate way to annotate viral genes, the experiments are often time-consuming, and involve huge costs to deal with such enormous data. Furthermore, wet experiments may miss some genes that are expressed only in some specific conditions with the limitation of laboratory techniques. Therefore, computational methods for viral gene prediction are needed to serve as assistance and reference instruments for experimental results. Currently, there are two major groups of computational methods to achieve relatively accurate viral gene prediction: the similarity-based methods and the *ab initio* methods. Z-curve is a type of widely applied theory in gene identification (Dong et al., 2016; Guo et al., 2017). Based on the Z-curve method, we developed ZCURVE_V in 2006, an *ab initio* gene finding software program for viruses, which has helped many researchers study virus genes over the past few years (Li et al., 2013; Huang et al., 2014; Mahony et al., 2015; Harrison et al., 2016).

In the present work, we updated and furthered the system based on ZCURVE_V (Guo and Zhang, 2006) by increasing the identifying variables for the classification model and adding a BLASTp searching module for gene predicting. Through these two modifications, the newly proposed Vgas system not only achieved higher prediction accuracy than ZCURVE_V but also provided functional gene annotations for predicted genes that are homologs to genes with known functions in public databases. As an application example of Vgas, 86 novel genes were found and assigned with explicit functions, while they were missed in RefSeq annotations. We believe that Vgas may help researchers to efficiently analyze unknown viral genomes.

## Materials and Methods

### The Implementation Process of Vgas

**Figure 1:**
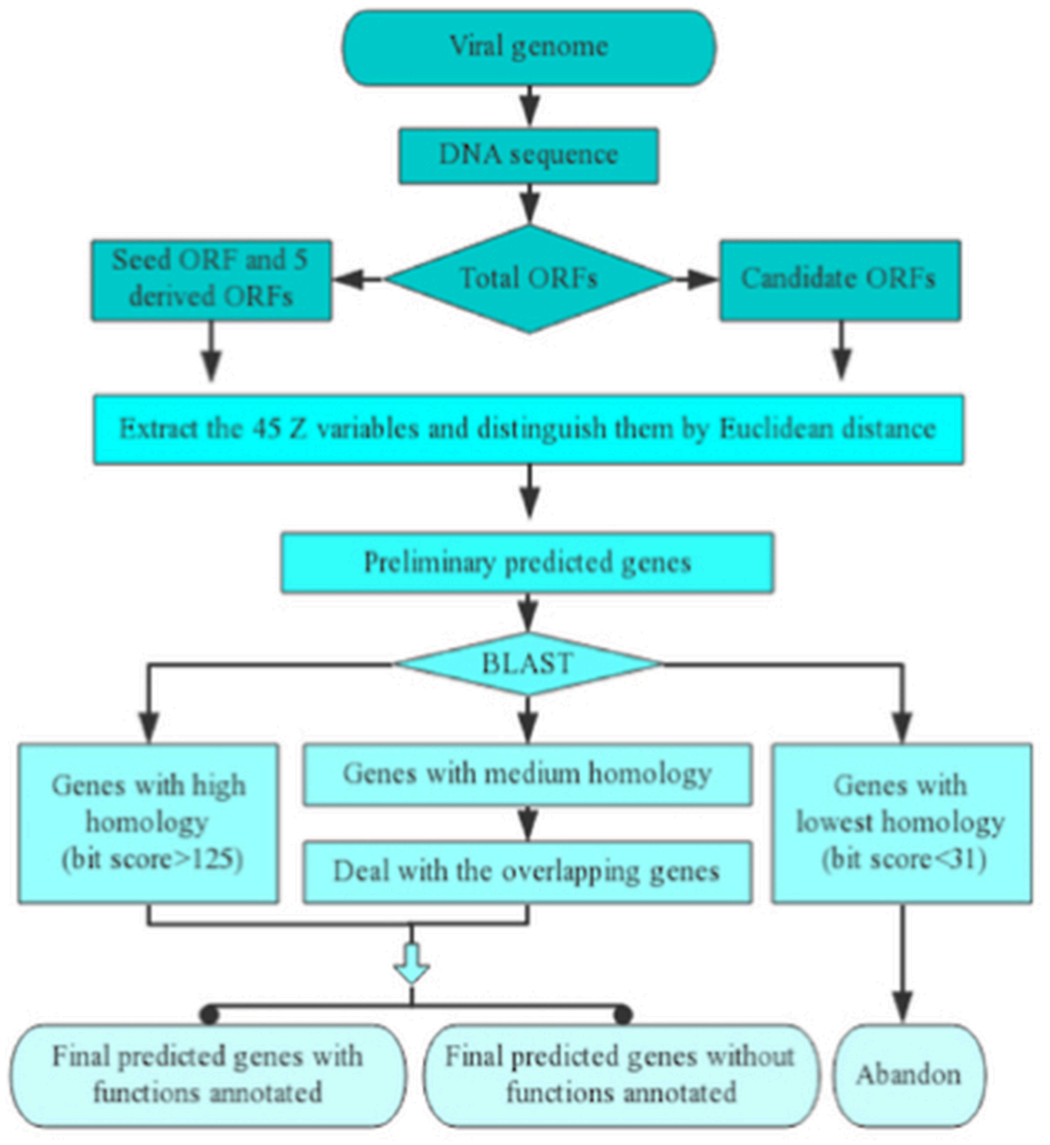
Implementation process of Vgas for one inputted viral genomic sequence. As shown, there are five successive steps.

The course of implementation of one inputted viral genomic sequence for Vgas processing can be divided into five successive steps (Figure 1). (1) Extracting all the ORFs from the genome sequence. (2) Finding the longest ORF as the seed ORF (representative of positive samples) and creating five derived ORFs (representatives of negative samples). Changing the phase position of the seed ORF will generate two derived ORFs, and changing the phase position of the complementary strand of the seed ORF can generate three additional ORFs. All of the five ORFs would be taken as representatives of negative samples. (3) Calculating the identifying variables and then distinguishing the ORFs by Euclidean distance discrimination to obtain the preliminarily predicted genes. If a candidate ORF has a closer distance with the seed ORF than all of the five artificial ORFs based on Euclidean distance, it will be predicted preliminarily as a gene; otherwise, it will not be predicted. (4) Performing a homologous search against the RefSeq database and determining the ultimately predicted genes. Because RefSeq contains all viral proteins stored in other databases, such as SwissProt, here, we only use it as a reference protein database. For some predictions that are homologous to genes with known functions (bit score > 150, e-value < 10^−40^), Vgas will transfer the functions of the latter to the predictions. In detail, Vgas will divide the preliminarily predicted genes into three groups according to the results of the BLASTp search against RefSeq viral genomes. One group of genes has the highest similarity to reference genes (bit score > 125, e-value < 0.01) and will be directly considered as the ultimately predicted genes. In contrast, some genes have the lowest similarity to reference genes (bit score < 31) and will be immediately eliminated. The remaining genes with medium similarity constitute the third group and will enter the next step. (5) Dealing with overlapping genes: these retained genes will be refined according to their overlapping ratios with longer genes. Consistent with ZCURVE_V, in comparing two overlapping ORFs, if the coding potential score of the longer ORF cut down by the given value is still higher than the shorter ORF, it will be recognized as a gene, and the shorter ORF will be considered non-coding. Otherwise, both ORFs are retained as coding ORFs. After this final step, all of the predicted genes can be determined.

It should be noted that we used only the 30 viruses listed in our previous work (Guo and Zhang, 2006) to set the parameters in our software; no other viruses were involved in the algorithm creation process.

Compared with eukaryotes and bacteria, there are a few characteristic properties for viral genes, and these differences will make it necessary to modify gene-finding algorithms devised for bacteria to fit viruses. On the one hand, viruses have folds-fewer gene numbers than cellular organisms. This feature makes it very difficult to choose large enough numbers of highly reliable seed ORFs. Without a number of training samples, it would be impossible to use machine-learning and other commonly used methods to construct classifying models. In Vgas, we chose the longest ORFs as the seed ORFs and used single center Euclidean discrimination to classify genes and negative samples. On the other hand, viral genes often tend to overlap each other, which makes it somewhat difficult differentiate true genes from those ORFs located in the shadow regions of true genes. High false-positive rates will appear if we choose to consider overlapping genes or ORFs. To deal with this problem, we generated five additional negative samples in the Vgas training set.

### Three Main Improved Features Compared With ZCURVE_V

To obtain higher sensitivity, we added 12 extra variables to ZCURVE_V; the original 33 variables are described as Equation (1):

(1) $$\{\begin{array}{l} x_{1},y_{1},z_{1}\\ x_{2},y_{2},z_{2}\\ x_{3},y_{3},z_{3} \end{array}\{\begin{array}{l} x_{12}^{A},y_{12}^{A},Z_{12}^{A}\\ x_{12}^{C},y_{12}^{C},Z_{12}^{C}\\ x_{12}^{G},y_{12}^{G},Z_{12}^{G}\\ x_{12}^{T},y_{12}^{T},Z_{12}^{T} \end{array}\{\begin{array}{l} x_{23}^{A},y_{23}^{A},z_{23}^{A}\\ x_{23}^{C},y_{23}^{C},z_{23}^{C}\\ x_{23}^{G},y_{23}^{G},Z_{12}^{G}\\ x_{23}^{T},y_{23}^{T},z_{23}^{T} \end{array}$$

where each of the three symbols, *x, y, z*, with subscripts and superscripts, denotes one variable, which can be the frequencies of the four nucleotides *A, C, G, T*, as in Equations (2, 3).

(2) $$\{\begin{array}{l} x_{i}=\left(a_{i}+g_{i}\right)-\left(c_{i}+t_{i}\right),\\ y_{i}=\left(a_{i}+c_{i}\right)-\left(g_{i}+t_{i}\right),\\ z_{i}=\left(a_{i}+t_{i}\right)-\left(g_{i}+c_{i}\right), \end{array}$$

(3) $$\begin{array}{l} \{\begin{array}{l} x_{k}^{x}=\left(p_{k}\left(XA\right)+p_{k}\left(XG\right)\right)-\left(p_{k}\left(XC\right)+p_{k}\left(XT\right)\right),\\ y_{k}^{x}=\left(p_{k}\left(XA\right)+p_{k}\left(XC\right)\right)-\left(p_{k}\left(XG\right)+p_{k}\left(XT\right)\right),\\ z_{k}^{x}=\left(p_{k}\left(XA\right)+p_{k}\left(XT\right)\right)-\left(p_{k}\left(XG\right)+p_{k}\left(XT\right)\right), \end{array}\\ X=A,C,G,Tk=12,23,31 \end{array}$$

In the above Equation (2), *i* denotes the codon position of one of the four mononucleotides located at a gene or negative sample, with a total of three codon positions. The lowercase *a, c, g*, and *t* with subscripts denote the frequency of each mononucleotide occurring at a given codon position. At each codon position, the sum of *a, c, g*, and *t* will be 1. Obviously, Equation (2) describes 9 total variables, which were transformed from the 12 frequency values of the four mononucleotides at three codon positions.

In the above Equation (3), *k* denotes the codon positions of one of 16 dinucleotides located at a gene or negative sample. At that time, we only considered short-distance association; codon position 31 was not involved because it corresponds to dinucleotides spanning two codons. The lowercase p denotes the frequency of one dinucleotide at a given codon position. There are a total of 32 frequency values for dinucleotides at 12 and 23 codon positions. With a similar transformation as used for Equation (2), these 32 values change to 24 variables.

In this work, we added another 12 variables, which are derived from 16 frequency values of dinucleotides at 31 codon positions, as in Equation (4):

(4) $$t_{i}\{\begin{array}{l} x_{31}^{A},y_{31}^{A},z_{31}^{A}\\ x_{31}^{C},y_{31}^{C},z_{31}^{C}\\ x_{31}^{G},y_{31}^{G},z_{31}^{G}\\ x_{31}^{T},y_{31}^{T},z_{31}^{T} \end{array}$$

We have now adopted 45 identifying variables instead of the 33 variables in ZCURVE_V 1.0 to represent a gene or negative sample sequence.

As the second improvement, we use five negative samples, which were all derived from the seed ORF. As an improvement over ZCURVE_V, two negative samples are derived via changing the phase position of the seed ORF. In addition, we obtained another three negative samples by changing the phase position of the complementary strand sequence of the seed ORF. In other words, the seed ORF contains six frames; the frame with the correct start and direction constitute the positive sample, whereas the other five frames are taken as negative samples. When calculating variable values, we excluded stop codons for all ORF sequences and positive/negative samples because five negative samples derived from the longest ORF would not adopt standard start and stop codons.

Third, we also utilize the BLASTp (Camacho et al., 2009) searching method to eliminate some false-positive predictions of hypothetical proteins and to assign functions to genes that are homologous to annotated genes with known functions. The E-value thresholds of the two operations are very different because one operation involves transferring functional information, while the other only involves deciding the coding potential. Please also note that we provide an option for predicting translation start sites. For this reason, we use the subprogram GS-finder to assign start sites for predicted genes. It has rather reliable predictions of translation start sites and correctly assigned 90% of 5 experimental sets of termini from *E*. *coli* and *B*. *subtilis* (Ou et al., 2004).

### Three Indexes Used to Evaluate Prediction Results

We used the following three indexes to evaluate our work:

(5) $$\begin{array}{rcl} precision & = & \frac{TP}{TP+FP} \end{array}$$

(6) $$\begin{array}{rcl} recall & = & \frac{TP}{TP+FN} \end{array}$$

(7) $$\begin{array}{rcl} F-score & = & \frac{2*precision*recall}{precision+recall} \end{array}$$

where the TP denotes the number of genes that were correctly predicted by the program, FP denotes the number of ORFs that were wrongly predicted as genes by the program, and FN denotes the number of genes that should be found by the program but were missed. If the translation terminal position of a prediction is consistent with the record in the database, we assume this annotated gene have been correctly predicted regardless of their overlapping ratio. Obviously, the higher these three indexes, the better the program performed. F-score, as a comprehensive index combining precision and recall, is a main standard reference for evaluating the performance of the software's prediction algorithm.

### Construction of the Benchmark Datasets

We used the RefSeq annotations of 5,705 viruses as the major benchmark to evaluate the prediction performance of four gene-finding programs. These viruses could be classified into five groups: ssDNA (830 viruses), dsDNA (2028), ssRNA (1629), dsRNA (809), and unknown (409); the viruses can subdivided into hundreds of families, with *Siphoviridae* (687) representing the largest family of our dataset. All strains of one virus will be tested if several strains are available. RefSeq's annotation may have bias to varied extents. However, no other datasets could be taken as benchmarks for large-scale measurements. To compensate for the drawback of RefSeq, we additionally constructed another benchmark of 100 viruses that have higher quality annotations. UniProtKB provides protein function annotations, and all entries correspond to validated or curated proteins. Based on this database, we could rank our 5,705 viruses in descending order of proportion of curated protein fraction. We then classified all viruses into 100 groups according to genome size. For viruses in the 100 groups, we chose those with the highest curated protein fraction as the representative of each group.

## Results and Discussion

### The Comparison Results Based on Different Test Sets

To perform an objective evaluation, we tested 5,705 viral genomes downloaded from RefSeq^1^ (in March 2017). During the process of constructing and training the Vgas prediction model, every virus involved was considered as an independent test set. We ran Vgas for each virus to obtain gene prediction results. In the BLASTp search procedure of the Vgas algorithm, we removed the query species itself from the reference database to avoid its unfair influence on the prediction.

We used the same dataset to test four other software programs as a comparison: ZCURVE_V, Prodigal (meta option) (Hyatt et al., 2012), GeneMarkS (Borodovsky and Lomsadze, 2014), and Glimmer (Delcher et al., 1999). The same indexes were used to measure the results. It should be noted that 1,041 viruses out of 5,705 viruses are so small that Glimmer could not achieve any gene prediction; therefore, we excluded this program from the assessment and only compared the performances of the other three programs.

In the practical process of bacterial gene annotation, several programs are often used to obtain collaborative predictions, which will usually be better than the predictions derived from individual programs (McHardy et al., 2004). In our previous work (Guo and Zhang, 2006), we also demonstrated that better results could be obtained for virus genomes when we jointly used ZCUVE_V and GeneMark. Here, we again test this strategy by combining Vgas, Prodigal, and GeneMarkS after running each of the programs individually. In the case of joint applications, if more than two software programs found the same gene, this gene was kept in the final result; otherwise (only one program predicted this gene), the gene was removed. As shown in Table 1, the general results support Vgas as the best prediction program, as it showed the highest values for all three measuring indexes among the three programs. Consistent with our inference, the joint strategy provided better predictions than only Vgas. We then divided all 5,705 viruses into four groups: phage, small genomes (≤ 10 kb), medium genomes (10–30 kb) and large genomes (>30 kb). In this case, Vgas' performance fell short for phages but still achieved the best index values for the other three groups. Especially in the small virus group, Vgas greatly surpassed the other two programs, with 6% higher precision, 2% higher recall, and a 5% higher F-score. Given that this group occupies most (3,514/5,705 = 61.6%) of the virus size profile, Vgas should be the preferred choice for practical annotation, particularly in the case of small-size viruses. Additionally, it is most sensible if Vgas is used jointly with Prodigal and/or GeneMarkS.

**Table 1:** The average prediction performance levels of Vgas, ZCUVE_V, Prodigal, and GeneMarkS for different genome sizes.

**Software**			**Vgas (%)**	**ZCURVE_V (%)**	**Prodigal (%)**	**GeneMarkS (%)**	**Combined (%)**
Other viruses
	All genomes (5705)	Precision	88.60	87.70	87.63	86.68	92.19
		Recall	92.22	81.06	91.10	87.97	93.68
		F-score	88.91	81.68	87.68	85.33	91.80
	Phages (1418)	Precision	80.87	86.16	93.61	92.12	93.14
		Recall	92.27	80.66	94.71	94.75	95.13
		F-score	85.60	82.87	93.84	93.07	93.80
	Small genomes	Precision	91.86	88.29	85.24	84.38	92.25
	(3514)	Recall	92.95	82.31	90.50	85.56	93.65
	(≤ 10 kb)	F-score	90.76	81.70	85.60	82.20	91.51
	Medium genomes	Precision	88.23	88.34	86.63	86.60	89.21
	(459)	Recall	87.89	75.09	86.95	85.62	90.97
	(10–30 kb)	F-score	85.78	77.47	84.78	84.17	88.51
	Large genomes	Precision	87.67	91.07	88.81	88.00	91.55
	(314)	Recall	90.00	77.53	87.60	87.81	91.49
	(>30 kb)	F-score	87.72	82.26	87.45	87.22	90.89

We also classified the dataset of 5,705 viruses into five groups including ssDNA (830 viruses), dsDNA (2028), ssRNA (1629), dsRNA (809), and unknown (409). The group of unknown represents those viruses that have not yet been classified into any of above other four groups in GenBank. As shown in Table 2, we can see that Vgas achieved the highest F-scores in the group for ssDNA (87.83%), ssRNA (91.13%), dsRNA (98.26%), and unknown (87.04%) among the four software programs; in ssDNA in particular, the performance of Vgas was markedly better than that of the other three. For the dsDNA group, although Vgas did not perform the best, the results were acceptable, and its performance was greatly improved over the original ZCURVE_V, with an F-score of 84.20% compared with 78.92%.

**Table 2:** The average prediction performance levels of Vgas, ZCUVE_V, Prodigal and GeneMarkS for different genome types.

**Software**		**Vgas (%)**	**ZCURVE_V (%)**	**Prodigal (%)**	**GeneMarkS (%)**	**Combined (%)**
ssDNA (830)	Precision	87.96	78.42	74.20	74.01	90.39
	Recall	90.90	79.76	88.68	85.77	91.65
	F-score	87.83	75.81	78.30	76.69	89.72
dsDNA (2028)	Precision	83.12	87.05	90.26	89.00	92.29
	Recall	88.75	75.62	89.97	90.16	92.53
	F-score	84.20	78.92	89.20	88.65	91.65
ssRNA (1629)	Precision	90.13	88.18	85.29	83.92	89.27
	Recall	95.30	83.87	92.74	86.18	95.37
	F-score	91.13	82.65	86.81	82.37	90.64
dsRNA (809)	Precision	98.67	98.28	97.87	97.87	98.72
	Recall	98.71	95.68	96.36	94.87	98.66
	F-score	98.26	95.92	96.37	95.32	98.35
Unknown (409)	Precision	91.09	89.50	90.84	89.78	94.02
	Recall	86.86	70.09	84.71	79.07	86.98
	F-score	87.04	74.90	85.17	78.48	88.42

For further confirmation of Vgas' performance, we repeated the above test based on a curated dataset with 100 viruses, instead of RefSeq. As shown in Table 3, Vgas obtained the highest F-score of 83.6%, higher than Prodigal and GeneMarkS (79.64 and 78.57%, respectively). Additionally, collaborative prediction was better than the use of any one software program.

**Table 3:** The average performance levels of Vgas, GeneMarkS and Prodigal for the curated viruses set.

**Software**	**Vgas (%)**	**GeneMark (%)**	**Prodigal (%)**	**Combined (%)**
Precision	79.08	73.00	73.28	78.96
Recall	93.82	90.00	93.15	94.53
F-score	83.60	78.57	79.64	84.24

### Comparison of the Annotation Function of Vgas With Other Software Programs

Some software programs are widely used for annotating prokaryotic genomes, such as Prokka (Seemann, 2014) and RAST (Aziz et al., 2008). The former uses Prodigal 2.6 and the latter uses Glimmer3 as their *ab initio* gene-finding programs. Hence, their performance for finding viral genes will be generally worse than Vgas, as shown in Tables 1–3. Like the two annotating systems, Vgas can also assign functions to genes that are highly similar to experimentally known genes referenced in public databases. After we checked a few widely studied viruses, such as HIV and HBV, it was found that Vgas could assign functional information for more genes than Prokka and RAST. Although these two systems could be used for virus gene annotation, they should be principally devised for bacteria. Therefore, their reference genome databases may not contain gene sequences from sequenced viruses. We believe that this deficiency may result in the inconsistent performance of the two systems compared with Vgas. In a nutshell, Vgas is more suitable for viral gene annotation.

### The Analysis of Predicted Novel Genes

Here, we comprehensively analyzed additional predicted genes by Vgas for 5,705 viruses. After performing BLASTp against the GenBank and RefSeq databases, we found that 86 identified predictions were highly similar to previously annotated genes (bit score > 150, *e*-value < 10^−40^), and the functions of these genes' products had been experimentally validated or reliably inferred. Information for all of the 86 newly predicted genes and their most similar genes from the RefSeq database are listed in Supplementary Table 1. Among them, 46 do not have any overlapping nucleotides with other genes in the same genome. Most of the remaining genes have sequence-overlapping ratios of < 10, indicating that they are predicted to be coding genes based on their coding potentials rather than falling in the shadow regions of longer genuine genes. Meanwhile, these genes are homologs of functionally validated genes, and it is believed that all or at least the majority of them truly encode proteins. Note that these 86 genes were newly identified by us and have not been previously reported in RefSeq. Furthermore, GeneMarkS missed 52 of the newly predicted genes, and Prodigal missed nine of them. Our identification of these genes would provide help for studies comparing related viruses. These results illustrate that Vgas could be used to find novel genes from previously annotated viral genomes, enriching the gene set.

## Conclusion

Vgas, as an improved version of ZCURVE_V, combines an *ab initio* method and a similarity-based method to automatically find viral genes and annotate the gene functions. Systematic tests illustrated that the program was competitive with extant programs, such as Prodigal and GeneMarkS. Vgas can also be jointly used with other programs to improve the performance of single gene finders. As an application example of the new system, 86 novel genes were identified and assigned explicit functions when we validated our program on 5,705 test viruses. We hope that Vgas could be an alternative virus gene finder to annotate new genomes or to reannotate extant genomes.

## Author Contributions

F-BG designed and coordinated this project, and revised the manuscript. Y-ZG programmed Vgas, and K-YZ checked the results. K-YZ and Y-ZG drafted the manuscript. CD improved the Vgas algorithm. M-ZD and SL took part in the data analyses. All the authors read and approved this manuscript.

### Conflict of Interest Statement

The authors declare that the research was conducted in the absence of any commercial or financial relationships that could be construed as a potential conflict of interest.

## Footnotes

^1^NCBI FTP Site for Viruses. Available online at: ftp://ftp.ncbi.nlm.nih.gov/genomes/Viruses/

## Supplementary Material

Supplementary Table 1 Details of the 86 novel genes identified by Vgas that were missed in RefSeq annotations.
